# Implementation and initial evaluation of falls risk reduction resources in a rural Native American Community

**DOI:** 10.1186/s40621-021-00359-1

**Published:** 2021-12-06

**Authors:** Kyle M. Knight

**Affiliations:** Indian Health Service, 5600 Fishers Lane, Rockville, MD 20857 USA

**Keywords:** Falls, Falls risk, Community clinic, Falls risk screening

## Abstract

**Background:**

Although falls are common and can cause serious injury to older adults, many health care facilities do not have falls prevention resources available. Falls prevention resources can reduce injury and mortality rates. Using the Centers for Disease Control and Prevention’s (CDC) Stopping Elderly Accidents, Deaths & Injuries (STEADI) model, a falls risk clinic was implemented in a rural Indian Health Service (IHS) facility.

**Methods:**

A Fall Risk Questionnaire was created and implemented into the Provider’s Electronic Health Records system interface to streamline provider screening and referral of patients who may be at risk for falls to a group falls risk reduction class.

**Results:**

Participants exhibited average improvements in the Timed Up and Go (6.8 s) (*P* = 0.0001), Five-Time Sit-to-Stand (5.1 s) (*P* = 0.0002), and Functional Reach (3.6 inches) (*P* = 1.0) tests as compared to their own baseline. Results were analyzed via paired *t* test. 71% of participants advanced out of an “increased risk for falls” category in at least one outcome measure. Of the participants to complete the clinic, all were successfully contacted and three (18%) reported one or more falls at the 90-day mark, of which one (6%) required a visit to the Emergency Department but did not require hospital admission.

**Conclusions:**

In regards to reducing falls in the community, per the CDC STEADI model, an integrated approach is best. All clinicians can play a part in reducing elder falls.

## Background

Coming of age beyond adulthood means different things to different cultures. The traditional notion of the respectful elder has been overtaken by the western obsession of youth. On many American Indian/Alaska Native (AI/AN) reservations, however, the former thought still prevails. Indigenous elders are vital to their respective societies (Viscogliosi et al. 2020). Elders are cultural gatekeepers and teachers of traditions, stories, and skills in nations that are trying desperately to remain intact (Viscogliosi et al. 2020).

**Fig. 1:**
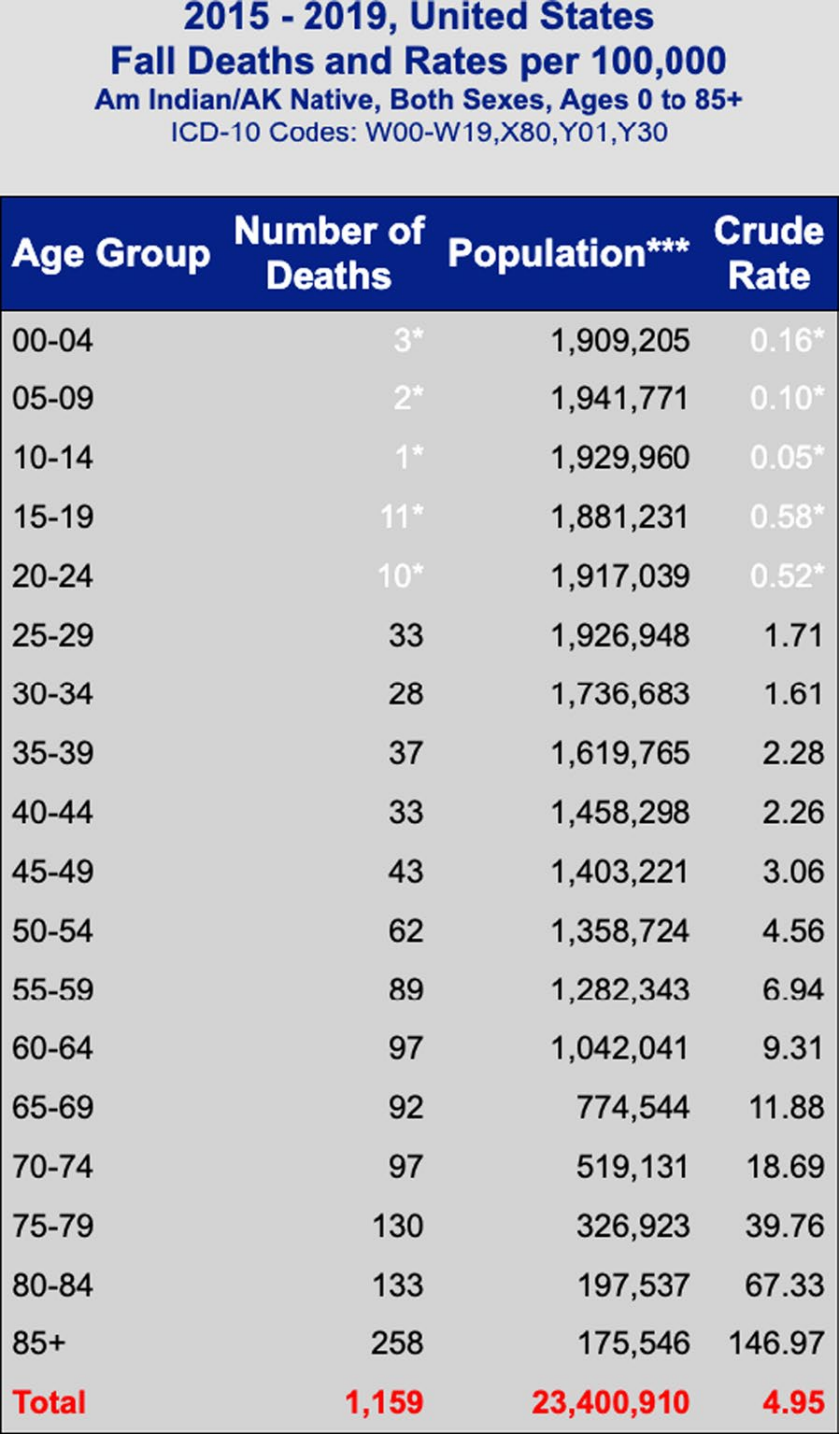
2015–2019 US fall deaths and rates per 100,000 [CDC]. *Rates based on 20 or fewer deaths may be unstable. Use with caution. **Standard Population is 2000, all races, both sexes. ***Population estimates are aggregated for multi-year reports to produce rates

“Unintentional injury” is the #7 cause of death in adults over 65 in the USA. 57% of the deaths in that category occurred by falling which make falling the leading cause of Unintentional Injury-related deaths for this age group (Centers for Disease Control and Prevention 2018a, b). If a fall is severe enough to warrant a hospital admission, the 1-year mortality rate of that individual is 50% (Klak et al. 2017). Many patients and health care personnel may also be under-informed as to the impact a fall can have on overall health, which may cause further complications bordering on iatrogenic (Hill et al. 2009; Shuman et al. 2016). During the most recent 5 year period, 1159 AI/ANs died due to a fall, with the death rates dramatically increasing from 9.31/100,000 population among 60–64 year olds to 67.33/100,000 among 80–84 year olds and 146.97/100,000 population among those 85+ years of age (Centers for Disease Control and Prevention 2021) (Fig. 1).

As compared with urban facilities, rural facilities are under-equipped, specialists are sparse, and medical services span larger geographical areas (Galambos 2005). In rural AI/AN communities, there are many low-income families and few own personal automobiles (Tiwari et al. 2015). Transportation issues are made worse by the poor road conditions that are found on many AI/AN reservations (Tiwari et al. 2015). These patients often have to rely on public transport or medical transit services that can be less than reliable (Tiwari et al. 2015).

In many instances, it is also possible for seemingly innocuous policies within health care companies to contribute to health disparities in rural populations. Though the Indian Health Service (IHS) does not operate in this way, it is not uncommon for private companies that provide home-health services to require their employees to meet a certain “productivity” obligation every pay period (Hellman 1991; Bly 1982). Productivity is mostly gained by treating patients but another way is the physical act of driving (Hellman 1991; Bly 1982). Once an employee hits a predetermined mileage quota, they may earn a production point in lieu of patient care. This means that for employees in rural areas who are driving further between homes (Galambos 2005), they are able to keep the same level of productivity while seeing fewer patients. As shown above, many different factors can lead to increasing health disparities between rural and urban health care.

This article examines the implementation and initial evaluation of a falls risk clinic, named the Community Health Injury Prevention (CHIP) Clinic, in an IHS facility. Many facilities lack a dedicated falls-related resource (Ayton et al. 2017), and the purpose of this article is to act as a scaffolding concept or a logistical framework in how to begin the implementation of such a resource. The Centers for Disease Control and Prevention (CDC) utilizes an evidence-based initiative called *Stopping Elderly Accidents, Deaths & Injuries* (STEADI) that seeks to reduce elder falls and injury (Centers for Disease Control and Prevention 2020). STEADI requires health care providers to Screen, Assess, and Intervene as part of the model (Centers for Disease Control and Prevention 2019, 2020). Because physicians, nurses, and pharmacists are likely to be the first point of contact in one’s care, they are crucial in their roles in the STEADI model, especially in regards to prescribing opioids, which are both frequently used by older adults and have been linked to increased falls (Ojha et al. 2014; Huang et al. 2012; Machado-Duque et al. 2018). Because Physical Therapists (PTs) spend, on average, more face-to-face time with patients than physicians and pharmacists (Murphy et al. 2005), they too are important to the STEADI process and can assume the brunt of the assessment and intervention role, while effective screening can be assumed by physicians in most medical models.

## Methods

### Screening

**Fig. 2:**
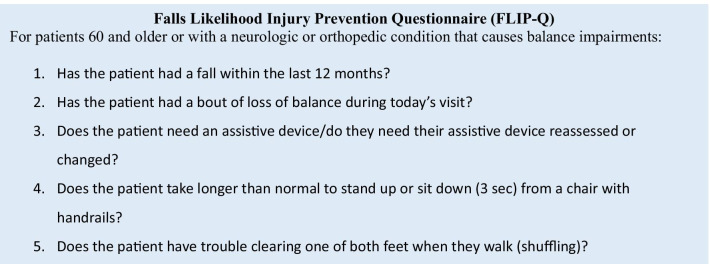
Falls Likelihood Injury Prevention Questionnaire (FLIP-Q)

A 5-item questionnaire, the Falls Likelihood Injury Prevention Questionnaire (FLIP-Q), was created for the falls risk clinic and uploaded to the facility’s Electronic Health Records (EHR) template. The questionnaire is as follows (Fig. 2) (Centers for Disease Control and Prevention 2019; Whitney et al. 2005; Nazarko 2006).

Each time a patient who meets the above criteria presents for a visit, providers are prompted to answer these five questions. An affirmative answer to any question will prompt a PT referral for a falls risk assessment and intervention, as described below.

### Assessment

The first of three group classes encompasses education, subjective history, assistive device assessment, completion of outcome measures, and instruction in a Home Exercise Program (HEP) which is to begin immediately and performed daily. Education consists of sharing mortality rates from falling, proper sitting/standing technique, proper footwear inside and outside the home, throw-rug/clutter removal in the home, and education on how pets might increase the risk for falls. The outcome measures performed in the first class are the Timed Up and Go (Whitney et al. 2005) (TUG) (dynamic balance test), the Five-Time Sit-to-Stand (Bohannon et al. 2007) (5xSTS) (lower-extremity strength test), and the Functional Reach (Weiner et al. 1992) (FR) (dynamic balance test).

### Intervention

During the second and third class, the HEP is performed. This allows a time for the clinicians (PTs/OTs) to assess compliance, as well as further promote correct form with all exercises. The second and third class also has participants complete higher-level dynamic balance exercises. At the beginning of the third class, the outcome measures are tested again and differences are noted.

The decisions made based on outcome measure performance use risk stratification levels as described by validated tests. Patients attending this class should be able to safely complete the TUG and 5xSTS in ≤ 15 s (Whitney et al. 2005; Bohannon et al. 2007) and should be able to score ≥ 7 inches on the FR (Weiner et al. 1992). Scores outside these safety thresholds result in an individual being placed into a higher risk for falls (Whitney et al. 2005; Bohannon et al. 2007; Weiner et al. 1992). If a patient is within the safety thresholds of all three measures, they are not compelled to complete the entirety of the clinic and are discharged. If a patient is within the “higher falls risk” category in any one of the outcome measures, they are considered appropriate for completion of the full CHIP class and only the improvement in measures in which they scored outside the safety threshold will be tracked. Therapists will make a decision on whether or not each patient will continue PT on an individual basis at the completion of the clinic.

Follow-up appointments or questionnaires are another vital component to comprehensive falls risk reduction clinics (Centers for Disease Control and Prevention 2019, 2020; Hauer et al. 2001; Shen et al. 2015). This CHIP clinic utilizes the following 90-day follow-up questionnaire: Have you had a fall in the last 90 days since the completion of the CHIP class? If yes, did this fall send you to the Emergency Department? If yes, were you admitted to a facility due to injuries resulting from the fall?

## Results

**Fig. 3:**
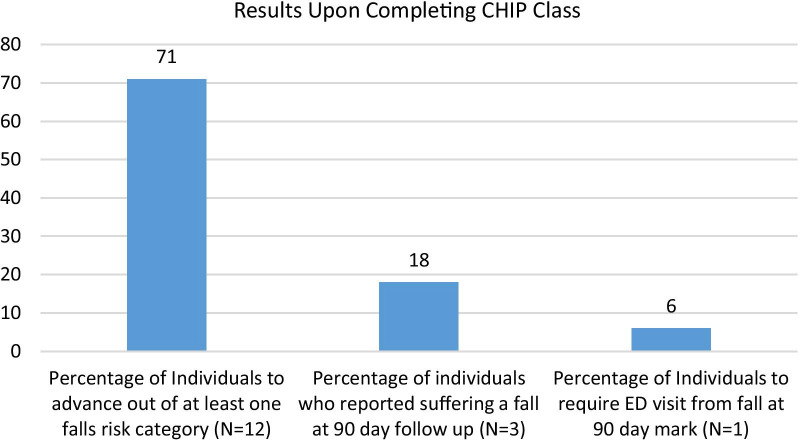
Percentage results after completing CHIP class

In the first 9 months of the CHIP clinic, providers screened over 2100 patients using the FLIP-Q. Of those who required further evaluation (*N* = 192) by the PT department, 17 completed all three classes; 165 people did not finish the entire clinic either because they scored within all safety thresholds or due to some other extenuating circumstance, 10 referrals were unable to be contacted via telephone or mail. Of the 17 participants who completed the program, there was an average TUG improvement of 6.8 s (*P* = 0.0001), an average 5xSTS improvement of 5.1 s (*P* = 0.0004), and an average FR improvement of 3.6″ (*P* = 1.0). Results were analyzed using a paired *t* test. 71% of participants advanced out of a “falls risk” category in at least one outcome measure. All participants were successfully contacted and three of the 17 patients (18%) reported falls at the 90-day mark, and one of these patients (6%) made a visit to the Emergency Department but did not require hospital admission (Table 1; Fig. 3).

**Table 1:** Average per-person improvement in outcome measures following completion of Community Health Injury Prevention (CHIP) class

Improvement following completion of CHIP class	
Outcome measure	Average improvement
Timed Up and Go Test	6.8 s (26%)
Five Times Sit-to-Stand Test	5.1 s (25%)
Functional Reach	3.6″ (106%)

Due to scoring within the safety threshold before completing any physical aspect of the class, three participants (14%) did not have their TUG tracked, four participants (19%) did not have their 5xSTS tracked, and 14 participants (82%) did not have their FR tracked. It is important to note that the only people completing this class in its entirety scored outside of the safety threshold in at least one of the three outcome measures at their initial visit (Table 2).

**Table 2:** Raw outcome measure data before and after CHIP class

Raw data CHIP class						
Patient	TUG Pre-CHIP	5xSTS Pre-CHIP	FR Pre-CHIP	TUG Post-CHIP	5xSTS Post-CHIP	FR Post-CHIP
1	24	18	7**	13	15	
2	18	17	7**	11	13	
3	22	22	3	12	12	8
4	24	29	8**	19	24	
5	25	18	7**	19	15	
6	16	16	8**	16	14	
7^+^	20	28	8**	21	13	
8	23	22	7**	13	11	
9*	10**	12**	10**	9	9	
10^+^	20	9**	7**	12		
11	67	30	4	44	32	6
12	22	15**	10**	16		
13	19	14	11**	15	13	
14	25	22	4	16	17	8
15^++^	23	21	7**	21	15	
16	17	16	7**	13	13	
17	35	36	8**	24	22	

*Though this patient scored outside of the falls risk criteria for all three tests, they exhibited unsafe bouts of loss of balance throughout so they were encouraged to complete the whole clinic

**Score is outside of falls risk category

^+^Patient reported fall at 90-day follow-up

^++^Patient reported going to the ED because of fall, but was not admitted to a facility

## Discussion

We found that two measures, the TUG and the 5xSTS, both improved significantly among older adults at risk for falls. The improvement in the FR was not determined to be statistically significant due to the small number of patients who were required to complete both FR tests. 82% of the participants who completed the class and scored outside of the safety threshold of the TUG and/or 5xSTS scored within the safety threshold of the FR. Based on these data, it appears that the FR is quite specific for increased falls risk, and reaching less than 7″ might be indicative of patients who are at a very high risk for falls.

There are limitations to note with this clinic, some of which stem from the fact that the population served by this clinic is rural and mostly low-income. Many of these patients are relying on medical transport, which may not get them to their appointment on time, if at all. There is also a fair number of patients living within the reservation served by this facility who only speak their native language (United States Census Bureau 2020). A 2011 study reported that only 78.8% of the Natives living in this area spoke English “very well.” (United States Census Bureau 2020) It is possible that some parts of the education from the CHIP class and the importance of consistent attendance are not communicated well due to the lack of specific native-language handouts. Though interpreters are used during the CHIP program, some of this information may be lost in translation, so to speak. This article is meant to serve as a template as to how and why one should get started in creating a falls prevention initiative in a facility that services a rural population. Though the sample size is small, the success of this clinic lies in its use of evidence-based tools and overall positive results.

## Conclusion

Following evidence-based practice is crucial when creating a new initiative of any type. In regards to reducing falls, per the CDC STEADI model, an integrated approach including screening, assessment, and intervention is best (Centers for Disease Control and Prevention 2020). All clinicians can play a part when working to reduce falls. It is also important to partner with as many appropriate team members as possible. This creates a pool of resources and ideas. These partners may include the admin board and IT department (they have the authority to approve additions to EHR templates; this was required with the implementation of the FLIP-Q), community health representatives to initiate home visits, environmental health personnel to assist with performing home-safety modifications, and existing community coalitions/programs or injury prevention initiatives that are already performing this work. All the aforementioned were required for the CHIP clinic. This clinic is a great example of how clinical staff and community-based fall prevention programs can work together to prevent falls.

Many Native American/Alaska Native cultures find importance in honoring the sacred and protecting those most vulnerable, including and especially their elders. Reducing elder falls can help families in AI/AN communities (and all rural communities) by freeing them from the burden of falls-related illness and injury. All clinicians can assist in this prevention effort and help rural and urban community members at higher risk for falls reduce injuries and live healthier lives.

## Data Availability

All data generated or analyzed during this study are included in this published article and its supplementary information files.

## Abbreviations

5xSTS: Five-Time Sit-to-Stand Test
CDC: Centers for Disease Control and Prevention
CHIP: Community Health Injury Prevention
FLIP-Q: Falls Likelihood Injury Prevention Questionnaire
FR: Functional Reach Test
HEP: Home Exercise Program
IHS: Indian Health Service
PT/PTs: Physical Therapy, Physical Therapist/Physical Therapists
STEADI: Stopping Elderly Accidents, Deaths, and Injuries
TUG: Timed Up and Go Test
USPHS: United States Public Health Service
